# Association of 25-hydroxy vitamin D with asthma and its severity in children: a case–control study

**DOI:** 10.1186/s12948-020-00122-9

**Published:** 2020-05-04

**Authors:** Alireza Sharif, Hamed Haddad Kashani, Mohammad Reza Sharif

**Affiliations:** 1 Infectious Diseases Research Center, Kashan University of Medical Sciences, Kashan, Iran; 2 Anatomical Sciences Research Center, Basic Sciences Research Institute, Kashan University of Medical Sciences, Kashan, Iran; 3 Gametogenesis Research Center, Kashan University of Medical Sciences, Kashan, Iran

**Keywords:** Asthma, Children, Health, 25-Hydroxy vitamin D, Patient

## Abstract

**Background:**

Universally, asthma has high prevalence rates and this has led numerous studies done into its causes. Despite extensive study on asthma the association between 25-Hydroxy Vitamin D (25(OH) vit. D) and asthma remains uncertain. In this study, the associations of 25(OH) vit. D levels with asthma and with the severity of asthma were evaluated.

**Methods:**

This was a case–control study performed in 2015 in the city of Isfahan. In this study 520 children were studied. Children with asthma were classified as cases and children who were referred for reasons other than respiratory problems and asthma were considered as controls. Serum 25 (OH) vit. D levels were then examined and compared between the two groups. Differences among groups were stated to be statistically significant when *P*-values < 0.05.

**Results:**

There were 260 asthmatic children and 260 controls in the present study. The mean 25 (OH) vit. D levels in the case group was 25.5 ± 16.62 and  16.76 ± 31.40 the control group and this difference was statistically significant (P < 0.05). 25(OH) vit. D levels were found to be 28.05 ± 16.98 in non-severe asthma and 21.41 ± 15.20 in severe asthma. Consequently 25(OH) vit. D level had inverse relationship with asthma severity (P = 0.002).

**Conclusions:**

As the results of this study showed, the lower level of 25(OH) vit. D correlated with the higher severity of asthma manifestations. Therefore, it is recommended that 25(OH) vit. D levels get routinely checked especially in severe asthma cases and if the deficiency presents, appropriate therapeutic measures be used to reduce the asthma severity.

## Background

Asthma is the most common chronic disease of childhood [1, 2] and is highly prevalent in industrialized countries [3]. This disease is caused by temporary obstruction of airflow due to the chronic inflammation of the airways and is identified by episodic and reversible attacks of wheezing, shallow breathing, shortness of breath and coughing [1]. Nowadays the number of children with asthma is rising so that in 2001 the percentage of patients with asthma had been 8.7% and this trends continues to point where according to CDC the number of people with asthma is anticipated to reach 400 million by 2025 [4]. The increasing prevalence of asthma has led extensive studies done into its causes. In recent years it has been suggested that vitamin D deficiency is associated with asthma [5–7] which could explain a significant portion of the increased incidence of this disease. While in normal people the need for vitamin D is supplied through sufficient exposure to sunlight and dietary intake [8–10], this vitamin deficiency has been recorded even in sun-replete areas of the world. Of course, diet and the geographic location can both affect this [11–14]. In a study in Europe, 93–97% of children in Denmark and Finland had vitamin D levels below 20 ng/mL [15], and in another study vitamin D deficiency was found in 95.4% of children [16]. Vitamin D plays an important role in bone metabolism and calcium maintenance. Many cells (brain, colon, prostate, breast, as well as immune cells) express vitamin D receptors which enables them to respond to 1, 25 dihydroxy vitamin D, the active form of the vitamin [17, 18]. For several reasons it is suggested that vitamin D plays an important role in immune response against infections [19, 20]. Most probably, vitamin D affects lung development and the function of immune system after birth [21, 22]. In a study was indicated that cord blood vitamin D insufficiency or deficiency in healthy neonates is associated with an increased risk of respiratory tract diseases [23]. Also, in one study it has been found that maternal vitamin D deficiency is associated with an increased risk of asthma in offspring [24] however, in another performed study no clear association has been shown between maternal vitamin D status and asthma in children [25]. In another study the cord blood vitamin D levels and its relationship to immune system function has been examined and no correlation has been found [26]. In some studies lower levels of vitamin D has been found in asthmatic patients that leads to decline in airway functions [27, 28]. Vitamin D supplementation in asthmatic patients can reduce the severity of the disease and can enhance airway responsiveness to glucocorticoids [29]. However, some other studies did not find an association between vitamin D and the airways function [30, 31]. With regard to the existing controversies we performed this study with the aim to examine the relationship of serum vitamin D levels with asthmatic state and severity of asthma.

## Methods and materials

In this study 520 children were examined with 260 children in the case and 260 children in the control group. 245 children were female and 275 were male. This case–control study was carried out on 5 to 15 years-old children referred to pediatrics clinics in 2015 in the city of Isfahan. The children with asthma were classified as cases and those who were referred for reasons other than respiratory problems and asthma were considered as controls. Demographic data of the study population were recorded. The diagnosis of asthma was based on clinical symptoms and spirometry findings and then the asthmatic patients were classified as mild, moderate and severe [1]. Despite treatment with high-dose inhaled corticosteroids (ICS), severe asthma in children is characterized by persistent symptoms. Children with severe asthma may fall into two categories, namely, difficult to treat asthma or severely treated resistant asthma. Asthma that is difficult to treat is defined as poor control due to poor diagnosis or comorbidity due to poor psychological or environmental factors [32]. In this study, the patients with asthma (the cases) were included into two subgroups based on the severity of their disease: the severe and the non-severe asthma (i.e. mild and moderate). These children were then enrolled after the confirmation of their disease by a pulmonary diseases or an asthma and allergy specialist. In this study, children with preexisting diseases of liver and kidney or endocrinopathies that could affect the levels of vitamin D were excluded. Participants who had a history of consumption of any supplements of vitamin D were excluded as well [33]. 3 mL of venous blood were collected from cases and controls and serum concentrations of 25(OH) vitamin D were assayed using standard ELISA test Kits (DIAsource ImmunoAssays Co.) [34, 35]. According to the guidelines outlined in the kit manual and the results of recent studies severe vitamin D deficiency was defined as vitamin D levels below 10 ng/mL, vitamin D levels between 10 and 30 ng/mL were considered as deficient, 30 to 100 ng/mL as normal and more than 100 ng/mL as toxic levels [36].

### Statistical methods

The Kolmogorov–Smirnov test was applied to determine the normal distribution of variables [37, 38]. The analyses were carried out based on the Intention-to-treat principle. These analyses were done using analysis of variance (ANOVA). We applied 1-way, but 2-tailed independent samples Student’s t tests and Chi square test for comparing the ratios between two groups. *P*-values < 0.05 were considered statistically significant. All statistical analyses were done using the Statistical Package for Social Science version 19 (SPSS Inc., Chicago, Illinois, USA).

## Results

The results indicated that the two groups were not significantly different in terms of the age and the gender of their participants but the mean 25(OH) vit. D concentrations in the asthmatic group were significantly lower than that of controls (P < 0.001). Details of these results are shown in Table 1.

**Table 1:** Groups in terms of age, gender and vitamin D levels

Variables	25(OH) vit. D		Age		Gender	
Groups	Standard deviation	Mean	Standard deviation	Mean	Female	Male
Asthma	16.62	25.57	2.82	9.53	117 (45%)	143 (55%)
Non-asthma	16.76	31.40	2.68	9.16	128 (49.2%)	132 (50.8%)
Total	16.93	28.49	2.75	9.35	245 (47.1%)	275 (52.9%)
*P*-value*	0.001		0.13		0.19	

*Statistical significance was attained when (*P*-value < 0.05)

40.8% of the asthmatic participants had normal levels of 25(OH) vit. D and the rest showed some degrees of 25(OH) vit. D deficiency while in the control group 55.4% of children had normal values. None of the subjects (neither the cases nor the controls) had toxic levels. On comparing serum 25(OH) vit. D levels the two groups were significantly different (P < 0.002). Table 2 shows the results in detail.

**Table 2:** The distribution of children according to vitamin D status in the studied groups

Variables	25(OH) vit. D		
Groups	Normal	Deficiency	Severe deficiency
Asthma	106 (40.8%)	105 (40.4%)	49 (18.8%)
Non-asthma	144 (55.4%)	86 (33.1%)	25 (11.5%)
*P-*value*	0.002		

*Statistical significance was attained when (*P*-value < 0.05)

Based on the spirometery 62.7% of cases had severe asthma and 37.3% were found to have non-severe asthma. The mean 25(OH) vit. D levels in the non-severe asthma subgroup were 28.05 ± 16.98 and 21.41 ± 15.20 in the severe asthma subgroup. There found to be a statistically significant association between levels of 25(OH) vit. D and asthma severity (P < 0.002).

Also in the severe asthma subgroup 68% and in the non-severe asthma subgroup 54% of cases had 25(OH) vit. D deficiency (values less than 30 ng/mL) to some degree. Table 3 shows the results in details.

**Table 3:** The association between vitamin D levels and asthma severity

Variables	25(OH) vit. D		
Asthma severity	Normal	Deficiency	Severe deficiency
Severe asthma	31 (32%)	40 (41.2%)	26 (26.8%)
None-severe asthma	75 (46%)	65 (39.9%)	23 (14.1%)
*P-*value*	0.001		

*Statistical significance was attained when (*P*-value < 0.05)

## Discussion

In our study 59.2% of the asthmatic patients and 44.6% of controls had 25(OH) vitamin D levels less than 30 ng/mL. In another study comparing the serum level of vitamin D in asthmatic patients and the control group 81% of cases and 64.1% of controls had vitamin D deficiency; these results are similar to ours [39] and considering the fact that the figures are high for both cases and controls in these two studies, it seems that a high percentage of the population in our country suffers from vitamin D deficiency [40]. Vitamin D deficiency is epidemic across the world and even in Persian Gulf littoral states where there is sufficient sunlight; vitamin D deficiency is highly prevalent. Studies have shown that severe vitamin D deficiency is found in more than 70% of teenage Iranian girls and above 80% of teenage girls in UAE [41]. In another study the vitamin deficiency was reported in 95.4% of elementary-school-age children (14). Although exposure to sunlight is a determinant factor for serum levels of vitamin D, the deficiency of this vitamin is highly prevalent even in sun-replete areas of the world [12]. Some possible explanations may include insufficient exposure to the sunlight as well as adhering to a diet lacking vitamin D.

In this study 25(OH) vitamin D levels in the asthmatic and none-asthmatic subjects were 25.57 and 31.40 ng/mL respectively and this difference was statistically significant (P < 0.05). Also in a study which was carried out on a sample of patients aged 6 months through 12 years hospitalized for acute respiratory illnesses, serum vitamin D levels in the patients with respiratory diseases was 26.8 ng/mL; which seems very similar to our results. However, in their study serum vitamin D levels was 26.1 ng/mL in the control group which did not differ from those with respiratory illnesses. In their study, of the entire cohort, 64.8% had vitamin D insufficiency; this may be due to choosing the control group from sick, hospitalized children which may have affected their results. In a case–control study of 263 subjects of ages 2–19 years with asthma who were compared to 284 non-asthmatic controls of similar ages, there was no difference in 25(OH)D between asthmatic patients and controls (28.64 vs. 28.42). In another study in Iran, serum levels of this vitamin was 18.92 ng/mL in asthmatic patients and 63.5% of cases had vitamin D deficiency while in the control group serum levels were 19.58 ng/mL and it was found that 53.3% of controls had vitamin D deficiency and the difference between these two groups was not significant [39].

In our study, 59.2% of the asthmatic subjects were vitamin D deficient which proves to be significantly higher than controls (44.6%). In Bener and colleagues study which was carried out on asthmatic children it was indicated that asthmatic children had significantly reduced serum vitamin D levels compared to non-asthmatic children; 68.1% of all asthmatics were vitamin D deficient [42]. In another research the figures were even higher and 91% of asthmatic patients had vitamin D levels below 20 ng/mL  [43]. Also others reported a higher percentage of vitamin D deficiency in asthmatic subjects compared to controls [44, 45]. These findings are consistent with our results. Brehm and colleagues study showed that vitamin D deficiency is less prevalent among asthmatic subjects compared with controls (44 vs. 47%) however this difference was not significant [46] which is inconsistent with our results. Various studies have used different definition for vitamin D deficiency which can affect the reported results. Also it should be taken into consideration that different studies have had various methods for selecting their controls. We cannot yet give an absolute answer to this question that whether vitamin D deficiency causes asthma or exacerbates its progress; but this vitamin has proven effects on pulmonary function [47].

In the current study serum vitamin D levels in the severe asthma subgroup was 21.41 and was 28.05 in the none-severe asthmatics and vitamin D levels were associated with asthma severity. Other study found no association between vitamin D and asthma severity [48, 49]. These are not consistent with our findings. In the study done by Gupta et al. which investigated the relationships between serum vitamin D, asthma severity and airway function, vitamin D was associated with asthma severity and pulmonary function and vitamin D supplementation had been useful in the treatment of pediatric severe therapy-resistant asthma [50]. Another study was found that vitamin D levels in children with asthma is associated with more severe disease [51]. Poon also has confirmed these results and believes that vitamin D deficiency leads to the development of severe asthma and if vitamin D deficiency is corrected, it will play a protective role against the progression of asthma and this may simply be the most effective therapy [52]. In Brehm’s study even though there was no significant different in terms of vitamin D deficiency between cases and controls; most patients with severe asthma had vitamin D deficiency which implicates that vitamin D deficiency is correlated with asthma severity [46]. Another study showed that vitamin D deficiency is associated with asthma severity [27]. Other research presented reduced serum levels of vitamin D were associated with increased asthma severity and increased hospitalizations and acute asthma attacks [53]. One explanation may be the fact that children with severe asthma, due to the severity of their disease, less frequently take part in physical activity and have reduced exercise and exposure to sun light which leads to vitamin D deficiency [43]. Several clinical and epidemiological studies have confirmed that vitamin D and corticosteroids may have a synergistic effect on asthma. Clinical studies have shown that vitamin D and corticosteroids exert a synergistic effect on asthma and other diseases to increase the anti-inflammatory effects of steroids [54]. Vitamin D has anti-inflammatory effects and enhances the responsiveness to corticosteroids especially in patients with corticosteroid-refractory asthma [55]. In the research of Yadav, supplementation of vitamin D significantly reduced the requirement of steroids and emergency visits and also vitamin D significantly reduced the level of severity of asthma patients over 6 months of treatment [56]. In the other study use of these supplements reduced the need for anti-asthma drugs [57]. According to Keating, this vitamin reduces inflammatory cytokines in some immune cells and co administered dexamethasone and vitamins D, will further reduce the production of inflammatory cytokines in asthma [58]. Vitamin D also regulates the capacity of immune cells to respond to allergens and thus can prevent allergies [59].

## Conclusion

In conclusion, we demonstrated reverse correlation of less serum vitamin D levels, with more severe the asthma manifestations. Thus it is recommended that serum vitamin D levels be routinely checked especially in severe asthma [60] and if its deficiency is confirmed, appropriate treatment should be provided so that the severity of asthma symptoms and the need for drugs are reduced.

## Data Availability

The dataset used in this study is available with the authors and can be made available upon request.

## Abbreviations

25(OH) vit D: 25-Hydroxy vitamin D
ANOVA: Analysis of variance
ELISA: Enzyme-linked immunosorbent assay
